# Psychostimulant prescribing trends in a paediatric population in Ireland: a national cohort study

**DOI:** 10.1186/s12887-015-0435-3

**Published:** 2015-09-10

**Authors:** Fiona Boland, Rose Galvin, Udo Reulbach, Nicola Motterlini, Dervla Kelly, Kathleen Bennett, Tom Fahey

**Affiliations:** HRB Centre for Primary Care Research, Department of General Practice, Royal College of Surgeons in Ireland, 123 St Stephens Green, Dublin 2, Ireland; Department of Clinical Therapies, University of Limerick, Castletroy, Limerick; Department of Public Health and Primary Care, Trinity College Centre for Health Sciences, Trinity College Dublin, Dublin, Ireland; Department of Pharmacology and Therapeutics, St James’s Hospital, Trinity College, Dublin, Ireland

**Keywords:** Children, Pharmacoepidemiology, ADHD, Psychostimulant treatment

## Abstract

**Background:**

Psychotropic paediatric prescribing trends are increasing internationally. The aim of this study is to examine the prevalence and secular trends in psychotropic prescribing in Irish children and adolescents between 2002 and 2011.

**Methods:**

Data was obtained from the Irish General Medical Services (GMS) scheme pharmacy claims database from the Health Service Executive Primary Care Reimbursement Services (HSE-PCRS). Prescribing rates per 1000 eligible population and associated 95 % confidence intervals (CIs) were calculated across years (2002–2011), age groups (0–4, 5–11, 12–15 years) and gender. Rates of concomitant prescriptions for psycholeptics and antidepressants were also examined. The total expenditure costs were calculated and expressed as a percentage of the cost of all prescriptions for this age group (≤15 years).

**Results:**

In 2002, 3.77/1000 GMS population (95 % CI: 3.53–4.01) received at least one psychostimulant prescription and this rate increased to 8.63/1000 GMS population (95 % CI: 8.34–8.92) in 2011. Methylphenidate was the most frequently prescribed psychostimulant. For both males and females the prevalence of medication use was highest among the 12–15 year old group. On average, a psycholeptic medication was prescribed to 8 % of all psychostimulant users and an antidepressant was concomitantly prescribed on average to 2 %. Total expenditure rose from €89,254 in 2002 to €1,532,016 in 2011.

**Conclusions:**

The rate and cost of psychostimulant prescribing among GMS children and adolescents in Ireland increased significantly between 2002 and 2011. Further research is necessary to assess the safety, efficacy and economic impact of concomitant psychotropic prescribing in this population.

## Background

Psychotropic paediatric prescribing is increasing in the USA, Europe and Australia [1, 2]. One of the main indications for psychotropic prescribing in children is the psychostimulant treatment of attention deficit hyperactivity disorder (ADHD) [3]. This disorder is defined by a combination of developmentally inappropriate and maladaptive levels of inattention, hyperactivity and impulsivity which persist for at least 6 months and are present in more than one social environment [4]. Epidemiologic studies using standardised diagnostic criteria suggest that 3 to 8 % of the school-aged population (elementary through high school) may suffer from ADHD [5, 6]. A recent systematic review examined ADHD prevalence estimates internationally by aggregating 154 prevalence studies of ADHD conducted from 1985 to 2012 [7]. The findings demonstrate that there is no evidence to suggest an increase in the number of children in the community who fulfil the criteria for ADHD when standardised diagnostic procedures are adhered to [7]. However, prescribing trends do not support this finding, where the prescription of psychostimulants has significantly increased in most countries worldwide for both pre-school and school-aged children [8–12].

Although ADHD is mainly treated with stimulants, a multimodal comprehensive treatment approach with individualised psychosocial interventions is recommended [4, 13]. One study assessing paediatricians’ and child psychiatrists’ professional approaches and attitudes to attention difficulties in the UK indicated that more than 60 % of both groups were prepared to prescribe stimulant medication without a formal diagnosis which is inconsistent with current prescribing guidelines [14]. Other studies have also highlighted wide regional variation in prescribing rates of psychostimulants [15, 16]. In terms of stimulant prescribing, methylphenidate and dexamfetamine are widely used in Europe and North America to reduce the symptoms of the condition [17]. Evidence from recent meta-analyses support their use with favourable effects demonstrated on measures of hyperactive, inattentive and impulsive behaviour [18, 19]. According to current European treatment guidelines [13] and licensing authorities, methylphenidate is considered off-label for children under 6 years of age and dexamfetamine use is not licensed for children under 3 years. Therefore caution is required in relation to their prescription in pre-school children [20]. A further prescribing challenge faced by clinicians treating children with ADHD relates to the prescribing of multiple medicines as these children are more likely to have a diagnosis of other neurobehavioral disorders and to receive non-stimulant psychotropic medications [4]. The benefit of a polypharmacy treatment strategy for children with ADHD is unclear and there remain questions regarding efficacy, safety and role of any combination therapy [21].

The aim of the current study is to determine the level of psychotropic prescribing in an Irish paediatric population (aged 0–15 years) receiving free medical care over a 10 year period (2002–2011), using dispensed medication data. Specifically, overall stimulant prescribing rates, types of stimulant drugs prescribed and overall cost of stimulant prescribing in Irish children are examined.

## Methods

### Study design

This was a national cohort study of children aged 0–15 years between January 2002 and December 2011. The STROBE standardised reporting guidelines were followed to ensure the standardised conduct and reporting of the research [22]. The methodology and design of this study are similar to a recently published study about antibiotic prescribing trends in a paediatric sub-population in Ireland [23].

### Data source and study population

Data was obtained from the Irish General Medical Services (GMS) scheme pharmacy claims database from the Health Service Executive Primary Care Reimbursement Services (HSE-PCRS) [24]. The GMS scheme is means tested and provides free health services to those who are unable to afford them. It represents approximately 28 % of Irish children but over-represents socially deprived populations [23]. The GMS database contains routinely collected data from pharmacy claims for dispensed medications. No information on diagnosis or disease condition is available.

The GMS database contains basic demographic information (age and sex) and all prescription items are coded using WHO’s Anatomical Therapeutic Chemical (ATC) classification [25]. Data is collected by the HSE-PCRS according to a number of pre-defined age groups. For this study, data was extracted for those aged 0–15 years (0–4, 5–11 and 12–15 years). The next age band (16–24 years) included a large proportion of adults and was therefore excluded from the current analysis. All psychostimulant medications classified according to the ATC system, were extracted for children aged ≤15 for the years 2002–2011. Relevant medication codes were: N06BA02, N06BA04, N06BA07 and N06BA09 [25]. For the purposes of our study, we defined consecutive users as children and adolescents who were prescribed a psychostimulant medication for ≥3 consecutive months during the study period. In the absence of information on diagnosis, this cut point was chosen to take into account children who may have been dispensed such a medication for a shorter period of time.

Concomitant psychotropic medication prescriptions were also extracted from the GMS database. All psycholeptic medications (ATC category N05, which includes antipsychotics (N05A), anxiolytics (N05B) and hypnotics and sedatives (N05C)) and additionally psychoanaleptic medications (i.e. antidepressants, ATC category N06A) were extracted.

Data was also obtained for the net ingredient cost (NIC) of each drug prescribed, which refers to the actual cost of the psychostimulant drugs. In addition, total expenditure was also calculated, which refers to the final cost to the Irish government to provide the medication for free and incorporates costs such as pharmacy fees and VAT in addition to the NIC.

### Ethical approval

Permission was given by the data controller to use the GMS dataset if anonymised and analysed at group level. Therefore, it was unnecessary to seek specific ethical approval for this study.

### Data analysis

Prescribing rates for all medications were compared across years, age groups (0–4, 5–11 and 12–15 years) and gender. The prescribing trends are described in terms of annual prescription rates of psychostimulants and are interpreted as the prevalence of children receiving at least one psychostimulant prescription per 1000 GMS population, as determined from the GMS database Prescribing rates per 1000 eligible population and associated 95 % confidence intervals (CIs) were calculated across years, age groups and gender. Additionally, annual NIC and the total expenditure costs were calculated and expressed as a percentage of the cost of all prescriptions for this age group (≤15 years).

A negative binomial regression model was used to determine trends in prescribing rates. The log of the GMS population was used as the offset term and year, age group, gender and all possible interactions between these variables were included in the model. The Bonferroni method was used to control the overall Type I error rate in making multiple comparisons of means. *P*-values <0.05 were deemed significant. Data analyses was performed using Stata version 11 (StataCorp, College Station, Tx, USA) and SAS version 9.3 (SAS Institute Inc. Cary, NC, USA).

## Results

### Population sample

During the study period (January 2002 to December 2011), the number of children ≤15 years in Ireland identified from the HSE-PCRS pharmacy database ranged between 188,833 and 311,579. On average, 51 % of the study population were male and 49 % were female.

### Prescribing trends

Table 1 displays the prevalence rate of psychostimulant prescribing (per 1000 GMS population) over the study period. In 2002, 3.77/1000 GMS population (95 % CI: 3.53–4.01) received at least one psychostimulant prescription and this rate increased to 8.63/1000 GMS population (95 % CI: 8.34–8.92) in 2011, representing a two-fold increase in psychostimulant prescribing over the study period. The prevalence increased each year except for 2006 where there was a non-significant decrease.

**Table 1:** Prescribing rates (95 % confidence intervals) and the cost of prescribing (% of all prescribing) psychostimulants to children aged 0–15 years from 2002 to 2011

Year	Rate of prescribing per 1000 GMS population (95 % confidence interval)	Net ingredient cost in € (% of all prescriptions)	Total expenditure in € (% of all prescriptions)
2002	3.77 (3.53–4.01)	68,945(0.70)	89,254 (0.64)
2003	4.91 (4.64–5.18)	236,180 (2.07)	268,810(1.67)
2004	5.67 (5.37–5.97)	351,204 (2.81)	393,406 (2.28)
2005	6.84 (6.51–7.17)	464,287 (3.34)	518,225 (2.75)
2006	6.77 (6.46–7.08)	529,926 (3.36)	596,176 (2.76)
2007	7.42 (7.10–7.74)	783,868 (4.42)	863,930 (3.59)
2008	7.61 (7.30–7.92)	940,133 (4.85)	1,036,285 (3.89)
2009	7.73 (7.43–8.03)	1,100,869 (5.10)	1,205,242 (4.00)
2010	7.80 (7.52–8.08)	1,279,381(5.33)	1,387,443 (4.12)
2011	8.63 (8.34–8.92)	1,410,443 (5.22)	1,532,016 (4.01)

**Fig. 1:**
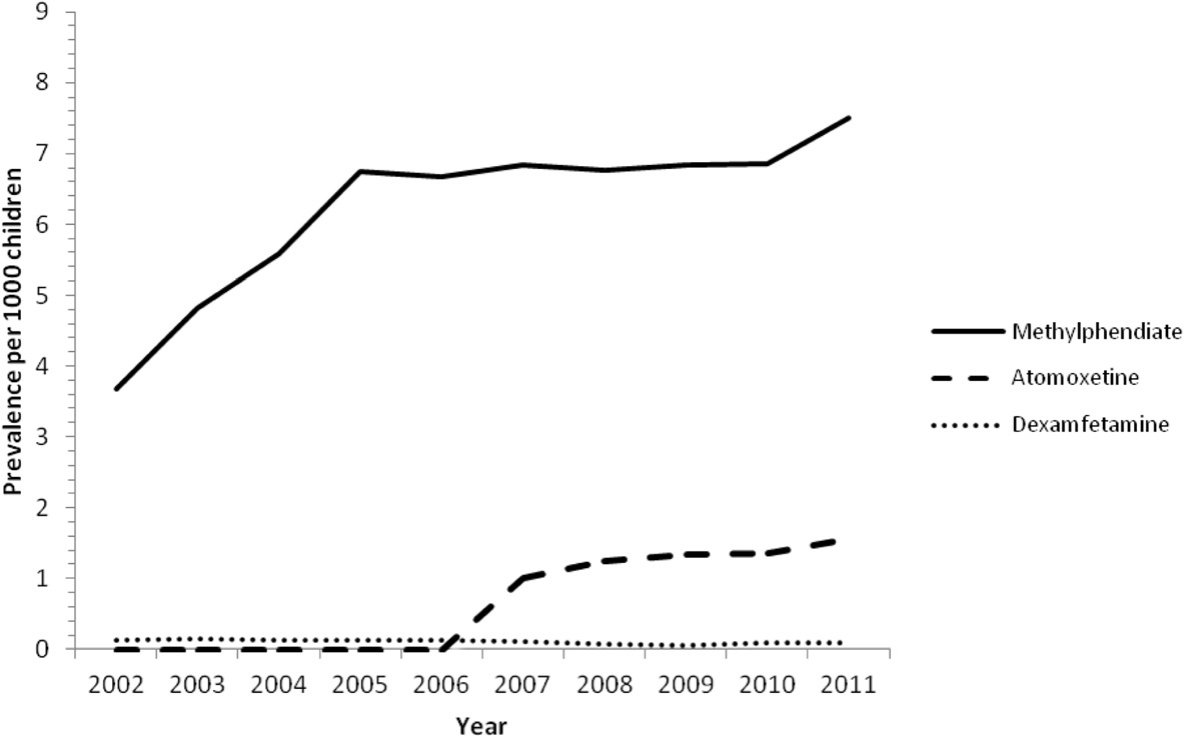
Prescribing rates of the three most frequently prescribed psychostimulants per 1000 GMS population aged 0–15 years old for 2002–2011

During the study period methylphenidate was the most frequently prescribed psychostimulant. The prevalence increased from 3.68/1000 GMS population (95 % CI: 3.44–3.92) in 2002 to 7.51/1000 GMS population (95 % CI: 7.24–7.78) in 2011. Rates of dexamfetamine remained relatively stable over the 10 years, fluctuating between 0.08/1000 GMS population (95 % CI: 0.05–0.11) and 0.15/1000 GMS population (95 % CI: 0.10–0.20). Atomoxetine became available for prescribing in Ireland in January 2007 and immediately became the second most commonly prescribed drug (1.00/1000 population, 95 % CI: 0.88–1.12). Since 2007 rates of atomoxetine have steadily increased to 1.57/1000 population (95 % CI: 1.45–1.70) in 2011. Figure 1 illustrates the prescribing rates of these drugs per 1000 GMS population over the study period. Modafinil was rarely prescribed between 2002 and 2011 with prevalences of less than 0.02/1000 GMS population.

### Gender and age

**Fig. 2:**
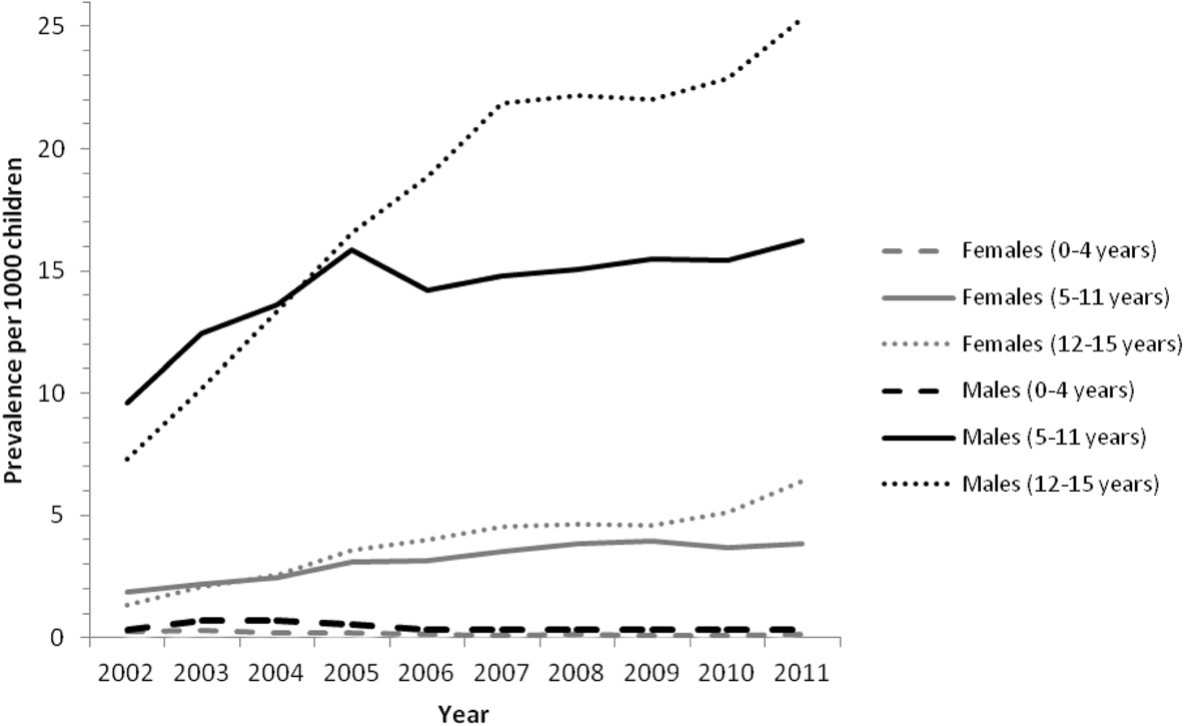
Prescribing rates of psychostimulants per 1000 GMS population aged 0–15 years old for 2002–2011 classified by gender and age group

Figure 2 displays the rates of prescribing classified by gender and age group during the study period. Statistically significant differences were found for both gender and age. In the 5–11 and 12–15 year age categories, male children were significantly more likely to receive a psychostimulant prescription than females across all years. The only significant difference between males and females for the 0–4 year old groups was for 2004. For males, from 2006 onwards, the 12–15 year old group was significantly more likely to receive a psychostimulant prescription than the 5–11 year old group. For females, there was no significant difference in prevalence between the 12–15 year old group and the 5–11 year old group until 2009. Since 2009, 12–15 year old females were more likely to receive a psychostimulant prescription than 5–11 year old females.

In all, 61 % of children were prescribed at least one psychostimulant medication for ≥3 consecutive months. The percentage was significantly higher in boys than in girls (63 % versus 54 %), and differed across age groups; it was 30 % in the 0–4 year olds, 63 % in the 5–10 year old group and 60 % in the 12–15 year old group.

### Concomitant medications

Table 2 displays the percentage of psychostimulant users taking a concomitant medication during the study period. On average, a psycholeptic medication was prescribed to 8 % of all psychostimulant users and an antidepressant was concomitantly prescribed on average to 2 %. The proportion of a psychotropic (psycholeptic or antidepressant) concomitant medication did not change significantly during the observation period between 2002 and 2011.

**Table 2:** Percentage of psychostimulant users (aged 0–15 years) from 2002 to 2011 taking concomitant medications

Year	Psycho-stimulant users (n)	Concomitant Psycholeptics (%)	Concomitant Anti-depressants (%)	Psycholeptics + Antidepressants (%)
2002	936	6.94	2.67	0.96
2003	1223	9.24	2.94	1.06
2004	1376	6.61	2.62	0.94
2005	1650	8.67	1.76	0.91
2006	1779	8.32	1.52	0.67
2007	2066	8.03	1.50	0.44
2008	2280	8.46	2.28	1.05
2009	2591	8.53	2.24	0.96
2010	2888	8.03	1.77	0.66
2011	3349	7.73	1.97	0.78

Legend Table 2: Percentage of psychostimulant users aged 0–15 years who also received concomitant psycholeptic (column 3) or antidepressant (column 4) medication from 2002 to 2011. Psycholeptic medication includes antipsychotics, anxiolytics and hypnotics and sedatives

### Cost

The net ingredient cost of psychostimulants increased from €68,945 in 2002 to €1,410,433 in 2011. Total expenditure (approximate cost to the State) rose from €89,254 in 2002 to €1,532,016 in 2011. This equates to an average yearly increase of approximately €160,000. Table 1 displays the cost of psychostimulant prescribing between 2002 and 2011.

## Discussion

### Statement of principal findings

This study found that the rate of psychostimulant prescribing among children and adolescents in Ireland increased from 2002 to 2011, with the exception of 2006 where a small decrease was noted. For both males and females the prevalence of medication use was highest among the 12–15 year old group and declined with younger age. The cost of prescribing also rose significantly with the total expenditure in 2011 over 17 times higher than in 2002. The use of concomitant medication remained stable during the study period.

### Results in the context of the current literature

Our results indicate that the prescribing prevalence of psychostimulants in Ireland is lower when compared to other countries. The highest observed rates during 2002–2011 are comparable with German data in 2000 [8], whereas prescribing in Netherlands is at least 1.5–2 times higher than in Ireland, and prescribing in USA at least 5 to 10 times higher [8]. In contrast, our findings are broadly similar to a recent population based study in the UK where the overall prevalence of psychostimulant prescribing increased over the 6 year study period (2002–2008) from 4.83 to 9.18 per 1000 children aged 6–12 years [26]. Methylphenidate was the most frequently prescribed psychostimulant in Ireland, which is consistent with findings from other studies [8, 17, 27, 28]. We found that boys aged between five and 15 years were more likely to be prescribed a psychostimulant medication than girls. Male to female treatment ratios from the literature are similar, ranging from about 3.4:1 to 5.8:1 [9, 28, 29]. In addition to a higher prevalence rate of ADHD in boys [30], the gender difference may also indicate a higher diagnosis and referral rate for boys which may be influenced by more disruptive or aggressive behaviour patterns in boys when compared to girls. Our results also demonstrate that from 2006 onwards, boys aged 12–15 years were significantly more likely to receive a psychostimulant prescription than the 5–11 year old group. However, the majority of boys in both age groups were prescribed at least one psychostimulant for three or more consecutive months. These findings are in contrast to those reported in the UK [26] and in the Netherlands [31] where the highest prevalence of psychostimulant prescribing was observed in younger boys. These differences in prescribing trends may reflect the differing healthcare access and referral pathways and prescribing practices in these countries.

Prescribing trends among girls aged 5–15 years remained stable between 2002 and 2009 in our study. This is in contrast to a UK study that found that the relative increase in prescribing patterns during the study period was lower in boys when compared to girls the same age-the increase in prevalence in girls aged 6–12 years was 2.1 fold compared to an increase of 1.9 fold for their male counterparts [26]. Similar trends were observed in Dutch study where the prevalence and incidence rates of psychostimulant prescribing were consistently higher among boys than girls from 2000 to 2007, however the largest increases were observed in girls over the study period [31].

Prescribing rates among pre-school children (0–4 years) were low in our study and did not significantly increase during the study period. This is in keeping with international literature where there are low levels of psychostimulant treatment in children under 6 years reported [27, 29]. Almost one third of children in this age category received a prescription for ≥3 months and methylphenidate was also the most commonly prescribed drug. These prescriptions are considered off-label as European guidelines [13] do not permit the prescription of methylphenidate for children under 6 years of age. In addition, safety and efficacy data for psychostimulant use in this age group is not available. However, our lack of information of indication for prescription limits the wider comparability of the results. Co-prescribing was most common with a psycholeptic medication (8 %) and less so, with anti-depressants (2 %). This differs slightly with the literature, which found stimulants were most commonly prescribed with anti-depressants [8, 28, 32] and alpha-agonists [8, 28]. Potential drug interactions between psycholeptics, particularly when used as anti-convulsants, and stimulants have been highlighted [20]. Few studies have evaluated the long-term safety of drugs for ADHD. A recent systematic review summarised the findings of eight clinical trials that examined the safety of medicines used for ADHD in children [33]. There was heterogeneity between the studies relating to data reporting and duration of follow-up (ranging from 1 to 4 years). However, the rate of treatment related adverse events (AE) ranged from 58 to 78 % in the included studies. The most common AEs included insomnia, headache, decreased appetite and abdominal pain. Furthermore, the rate of discontinuation ranged from 8 to 25 % due to AEs [33], highlighting the need for systematic monitoring of long-term safety of prescribing.

An increasing cost to the state due to psychostimulant prescribing was noted in our study, with expenditure in 2011 at approximately €1.5 million representing an average annual increase of approximately €160,000. The rising costs can most likely be attributed to the prescribing trend and rising drug costs. It is worth noting the increased cost of the long acting formulations compared to the immediate release formulations [13], the former being associated with improved compliance enhancing treatment in certain patient groups.

Changes in licensing and regulation should be considered when interpreting this data. The timescale of the study coincides with the revocation of the Irish Medical Preparations (Control of Amphetamines) Regulations and the licensing of more expensive controlled release preparations of methylphenidate and atomoxetine.

### Strengths and weaknesses of the study

This study examined prescribing trends in Irish children, aged 0–15 years, over a 10 year period from 2002 to 2011 using data from a national prescribing database. However, the findings from our study need to be interpreted in the context of the study limitations. A significant drawback of the study is that our database only contains routinely collected data for pharmacy dispensed medications with no information on diagnosis or indication for prescription. In the absence of this information, we examined duration of prescription to take into account children who may have been dispensed a psychostimulant medication for a shorter period of time. Secondly, we have not examined the dosage form or quantity of medication dispenses per prescription. Therefore the results of this paper may not accurately reflect prescribing patterns among patients with a clinical diagnosis of ADHD. Thirdly, our prescribing figures cannot be used as an estimate for the entire Irish paediatric population as study participants were means tested which results in an over-representation of lower socioeconomic groups [34, 35]. This may result in an over-inflation of the true prescription rates for Irish children; given that children from more socially deprived backgrounds are more likely to receive a psychostimulant prescription than their better off counter-parts [16]. Analysis of prescribing data among children and adolescents with private healthcare insurance in Ireland would facilitate more robust conclusions regarding the role of socioeconomic status on prescribing patterns of psychostimulants in the Irish context. Caution is also required when comparing findings with results from other countries, as in addition to methodological differences of studies, different licensing and reimbursement policies for medication may exist. Furthermore, no data was available on the clinical indication for treatment and professional details (specialist versus generalist) of the doctor initiating the prescription.

### Clinical implications and areas for further research

The management of children and adolescents with ADHD is a complex process. To this end, clinicians should evaluate the need for prescribing in this population on a case-by-case basis, taking into account the severity of the symptoms and the risk-benefit profile for different treatment alternatives, as per the current guidelines [36]. However, the longer-term safety of many medications used concomitantly with stimulants is largely unknown. Therefore, further research is necessary to assess the risk-benefit profile of concomitant psychotropic medication use in children with ADHD [36]. Geographical variation in psychostimulant prescribing has been also been reported in other studies and warrants further research [15, 16]. Furthermore, psychostimulant prescribing in additional age categories, particularly the 16–24 year old group, should be explored to gain an insight into prescribing patterns in early adulthood. Finally, previous studies have also reported that psychiatrists are more likely to prescribe psychotropic medications in this population than primary care physicians, highlighting the need for further investigation into variation in prescribing practices.

## Conclusion

The rate of psychostimulant prescribing among GMS children and adolescents in Ireland increased between 2002 and 2011. The use of concomitant medication remained stable during the study period. Though increasing, Irish prescribing of psychostimulants is modest when compared to the US and other European countries. Further research is necessary to assess the long-term efficacy, safety, and economic impact of concomitant psychotropic prescribing in this population.

## Abbreviations

ADHD: Attention deficit hyperactivity disorder
AE: Adverse events
GMS scheme: General Medical Services scheme
HSE-PCRS: Health Service Executive Primary Care Reimbursement Services
NIC: Net ingredient cost
WHO ATC classification: World Health Organisation Anatomical Therapeutic Chemical classification
95% CI: 95 % confidence interval
